# 巨球蛋白血症患者临床特征和预后并与Pivotal研究比较

**DOI:** 10.3760/cma.j.issn.0253-2727.2021.12.005

**Published:** 2021-12

**Authors:** 怡 陶, 硕 王, 黎 王, 敏 武, 维莅 赵

**Affiliations:** 上海血液学研究所，医学基因组学国家重点实验室，国家转化医学中心（上海），上海交通大学医学院附属瑞金医院，上海 200025 Shanghai Institute of Hematology, State Key Laboratory of Medical Genomics, National Research Center for Translational Medicine at Shanghai, Ruijin Hospital Affiliated to Shanghai Jiao Tong University School of Medicine, Shanghai 200025, China

**Keywords:** 巨球蛋白血症, 基因，MYD88, 布鲁顿酪氨酸激酶, 总体生存, 无进展生存, Waldenström macroglobulinemia, Gene, MY88, Bruton's tyrosine kinase, Overall survival, Progression-free survival

## Abstract

**目的:**

总结上海交通大学医学院附属瑞金医院108例华氏巨球蛋白血症（WM）患者的临床特征和预后，并与应用BTK抑制剂（BTKi）单药的Pivotal研究比较。

**方法:**

回顾性分析2008年3月至2021年2月收治的症状性WM患者的临床特征、无进展生存（PFS）和总体生存（OS），其中52例患者进行MYD88突变检测。

**结果:**

108例患者中位年龄63（38～78）岁，男女比例3.5∶1。IPSS评分高危组43例（40％），中危组36例（33％），低危组29例（27％）。与Pivotal研究63例患者的基线特征比较，年龄、性别、IPSS危险度、血清IgM水平、PLT的差异无统计学意义，但HGB（86 g/L对105 g/L）、血清β_2_-微球蛋白（3.1 mg/L对3.9 mg/L）、骨髓受累（13％对60％）、淋巴结肿大比例（41％对59％）低于Pivotal研究患者，脾肿大患者比例（27％对11％）高于Pivotal研究，差异均有统计学意义（*P*值均<0.05）。MYD88突变整体检测阳性率77％。中位随访36（1～121）个月，中位OS时间为95个月，中位PFS时间为35个月。患者2年OS率（83％对96％）和5年OS率（67％对87％）低于Pivotal研究。2008-2021年，以BTKi、CD20单抗和蛋白酶体抑制剂治疗为主的患者比例从50％逐步提升至93％，2015-2021年诊断患者的长期OS较2008-2014年改善（*P*＝0.048）。

**结论:**

包括BTKi在内的新药治疗使WM患者获益并改善其生存，中国WM患者MYD88和CXCR4突变检测阳性率及对BTKi的敏感性值得进一步探索。

## Abstract (en)

**Objective:**

This study aimed to summarize the clinical characteristics and prognosis of 108 patients with Waldenström macroglobulinemia（WM）in a single center and compare them with those of the Pivotal study with Bruton's tyrosine kinase inhibitor（BTKi）monotherapy.

**Methods:**

The clinical characteristics, international prognostic index score（IPSS）, first-line treatment, progression-free survival（PFS）, and overall survival（OS）of 108 patients with newly diagnosed WM from March 2008 to February 2021 were retrospectively evaluated. The MYD88 mutation was tested among 52 patients.

**Results:**

The median age of the 108 patients was 63 years（range, 38-78 years）with a male-to-female ratio of 3.5∶1. According to IPSS, we included 40％（*n*＝43）high-risk, 33％（*n*＝36）intermediate-risk, and 27％（*n*＝29）low-risk patients. In this study, no significant difference was observed in age, gender, IPSS risk, serum immunoglobulin M（IgM）, and platelet counts compared to the baseline characteristics of 63 patients in the pivotal study. However, hemoglobin（86 g/L *vs* 105 g/L）, serum β_2_-MG（3.1 mg/L *vs* 3.9 mg/L）, bone marrow involvement（13％ *vs* 60％）, and the proportion of adenopathy（41％ *vs* 59％）were significantly lower than those in the Pivotal group. The proportion of patients with splenomegaly（27％ *vs* 11％）was significantly higher than that of the Pivotal group. All the differences were statistically different（all *P* values <0.05）. The overall positive rate of MYD88 mutation was 77％. With a median follow-up of 36（1–121）months, the median OS was 95 months, and the median PFS was 35 months. The 2-year OS rate（83％ *vs* 96％）and 5-year OS rate（67％ *vs* 87％）of patients with WM in our center were lower than those in the Pivotal group. The proportion of novel treatments based on BTKi, CD20 monoclonal antibody, and proteasome inhibitors in our center from 2008 to 2021 has gradually increased from 50％ to 93％. The long-term OS of patients diagnosed in 2015–2021 was improved compared with that in 2008–2014（*P*＝0.048）.

**Conclusion:**

Novel drugs, including BTKi, continue to benefit patients with WM and improve their survival. It is worthwhile to further explore the positivity of MYD88 and CXCR4 mutations in the Chinese population and their sensitivity to BTKi.

华氏巨球蛋白血症（Waldenström macroglobulinemia，WM）是一种分泌IgM的淋巴浆细胞淋巴瘤，在非霍奇金淋巴瘤中占2％[1]。WM是惰性淋巴瘤，但大多数患者最终进展，需要新药改善其预后。文献报道90％的WM患者MYD88^L265P^突变[2]，而MYD88突变能通过布鲁顿酪氨酸激酶（BTK）活化NF-κB促进WM细胞生长[3]。口服靶向药物BTK抑制剂（BTKi）在WM患者中疗效突出，已在多个临床研究中得到证实[4]–[6]。2015年Treon等[4]首次报道63例经治WM患者应用BTKi单药的Pivotal临床研究，随访上述患者5年后，2021年发表了患者的长期生存结果[7]。WM发病率低，国内尚无研究报道真实世界WM患者的长期生存结果与临床研究患者的差异。本文旨在比较本中心WM患者与Pivotal研究患者基线临床特征和长期预后的差异，探讨患者应用新药的潜在长期生存获益。

## 病例与方法

1. 一般资料：回顾性分析2008年3月至2021年2月我中心收治的120例WM患者的临床资料，12例患者未达到治疗指征，收集108例患者的完整基线临床资料，包括性别、年龄、血常规、生化常规、免疫固定电泳、IgM水平、一线治疗方案、进展时间、生存时间等。所有患者均符合以下诊断标准：①血清中存在单克隆IgM（不论数量）；②病理检查证实骨髓中存在淋巴浆细胞浸润；③除外其他已知类型的淋巴瘤[8]–[9]。骨髓受累程度通过骨髓流式细胞术检测CD19^+^CD5^−^CD10^−^且轻链限制性表达的单克隆淋巴浆细胞比例表示。

2. 危险分层和疾病进展的定义：采用WM国际预后评分系统（ISSWM）进行危险分层[10]。该预后系统纳入5个危险因素：年龄>65 岁，HGB≤115 g/L，PLT≤100×10^9^/L，β_2_-微球蛋白（β_2_-MG）>3 mg/L，IgM>70 g/L。低危组：0或1个危险因素且年龄≤65岁；中危组：2个危险因素或年龄>65岁；高危组：2个以上危险因素。采用第7届国际WM工作组（IWWM）制定的疗效评估标准定义疾病进展[11]。

3. MYD88^L265P^突变检测：52例患者进行了MYD88检测，标本来源包括淋巴结、骨髓和外周血，检测方法包括等位基因PCR（AS-PCR）、数字PCR（qPCR）、一代测序（Sanger测序）和二代测序（NGS）。

4. 一线治疗方案：108例患者接受系统治疗，26例（24％）患者一线接受以BTKi为主的靶向治疗，其中22例应用BTKi单药，4例应用BTKi联合利妥昔单抗；54例（50％）患者一线接受以利妥昔单抗为主的免疫化疗方案，包括18例应用RCD（利妥昔单抗+环磷酰胺+地塞米松）方案，16例应用RCHOP（利妥昔单抗+环磷酰胺+阿霉素+长春新碱+泼尼松）/RCOP（利妥昔单抗+环磷酰胺+长春新碱+泼尼松）方案，14例应用RFC（利妥昔单抗+氟达拉滨+环磷酰胺）/RFMC（利妥昔单抗+氟达拉滨+米托蒽醌+环磷酰胺）方案，2例患者应用利妥昔单抗单药方案，2例患者应用BR（利妥昔单抗+苯达莫司汀）方案，2例患者应用R2（利妥昔单抗+来那度胺）方案；4例（4％）患者一线接受以硼替佐米为主的方案，包括2例PAD（硼替佐米+阿霉素+地塞米松）方案、2例RVD（利妥昔单抗+硼替佐米+地塞米松）方案；24例（22％）患者接受烷化剂或核苷酸类似物方案，包括CHOP（环磷酰胺+阿霉素+长春新碱+泼尼松）方案、FC（氟达拉滨+环磷酰胺）方案、TD（沙利度胺+地塞米松）方案或单用苯丁酸氮芥。将BTKi、利妥昔单抗和蛋白酶体抑制剂为主的方案定义为新治疗方案，其他方案定义为传统治疗方案。

5. 随访：通过查看门诊记录、住院病历及电话随访至2021年4月9日。无进展生存（PFS）时间定义为患者启动一线治疗至疾病进展或死亡的时间，总生存（OS）时间定义为患者启动一线治疗至死亡或末次随访的时间。

6. 统计学处理：采用 SPSS 23.0 软件进行统计学分析。组间率的比较采用似然比*χ*^2^检验，Kaplan-Meier法用于分析患者的生存情况，*P*<0.05为差异有统计学意义。

## 结果

1. MYD88突变阳性率：采集52例患者的外周血、骨髓或淋巴结等标本，通过AS-PCR、qPCR、Sanger测序或NGS方法检测出40例患者MYD88突变阳性，整体MYD88突变检测阳性率77％，其中39例患者MYD88^L265P^突变，1例患者MYD88^L273P^突变。52例患者中32例患者进行NGS检测，28例MYD88突变阳性，MYD88突变阳性率88％，5例CXCR4突变阳性，CXCR4突变阳性率16％。因部分患者同时进行了多标本检测，因此MYD88共检测91例次。以检测方法分类，AS-PCR检测28例次，MYD88阳性率75％；qPCR检测4例次，MYD88阳性率64％；Sanger测序检测13例次，MYD88阳性率46％；NGS检测36例次，MYD88阳性率86％。

2. 临床特征并与Pivotal研究比较：本中心108例WM患者基线临床特征见表1。与Pivotal研究纳入的63例患者基线特征比较，年龄、性别、IPSS危险度、血清IgM值、PLT的差异无统计学意义；本研究患者HGB、血清β_2_-MG、骨髓受累、淋巴结肿大比例低于Pivotal研究患者，脾肿大患者比例高于Pivotal研究。

**表1:** 本中心108例华氏巨球蛋白血症患者与63例Pivotal研究患者的临床特征比较

临床特征	本中心（108例）	Pivotal 研究（63例）	*χ*^2^值	*P*值
年龄［岁，*M*（范围）］	63（38～78）	63（44～86）	NA	NA
年龄>65岁［例（％）］	42（39）	–	–	–
性别［例（％）］			0.057	0.811
男	84（78）	48（76）		
女	24（22）	15（24）		
IPSS评分［例（％）］			1.569	0.456
低危	29（27）	14（22）		
中危	36（33）	27（43）		
高危	43（40）	22（35）		
IgM［g/L，*M*（范围）］	39.45（4.02～117.74）	35.20（7.24～83.90）	NA	NA
IgM>40 g/L［例（％）］	50（47）	26（41）	0.556	0.456
HGB［g/L，*M*（范围）］	86（39～163）	105（82～138）	NA	NA
<110 g/L［例（％）］	86（80）	37（59）	8.608	0.003
<100 g/L［例（％）］	72（67）	25（40）	11.802	0.001
PLT［×10^9^/L，*M*（范围）］	165（9～398）	214（24～459）	NA	NA
<100×10^9^/L［例（％）］	23（21）	7（11）	2.853	0.091
血清β_2_-微球蛋白［mg/L，*M*（范围）］	3.1（1.1～11.6）	3.9（1.3～14.2）	NA	NA
>3 mg/L［例（％）］	58（55）	45（71）	4.636	0.031
>3.5 mg/L［例（％）］	40（38）	35（56）	5.083	0.024
骨髓受累程度［％，*M*（范围）］	13（2～67）	60（3～95）	NA	NA
髓外受累［例（％）］				
淋巴结最大直径>1.5 cm	42（41）	37（59）	4.809	0.028
脾脏长径>15 cm	27（27）	7（11）	5.761	0.016

注：NA：不适用；–：无数据

3. 患者生存预后比较：本中心中位随访36（1～121）个月，中位OS时间为95个月，中位PFS时间为35个月。本中心患者的2年OS率（83％对96％）和5年OS率（67％对87％）均低于Pivotal研究，2年PFS率（46％对69％）和5年PFS率（28％对54％）也均低于Pivotal研究（图1）。

**图1:**
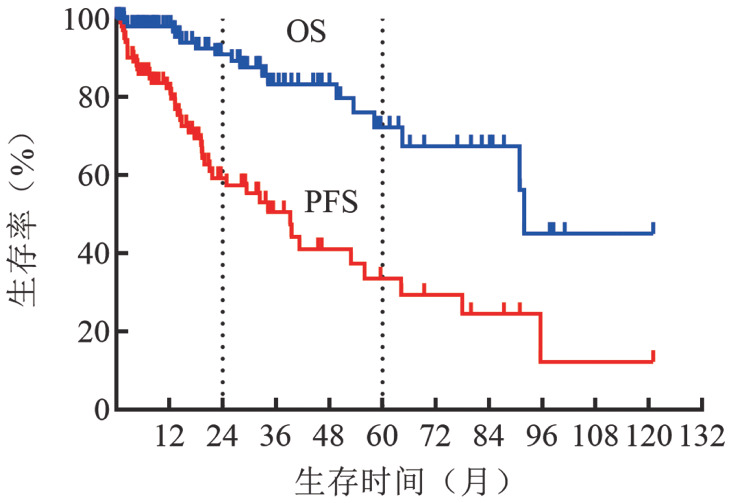
108例华氏巨球蛋白血症患者的总生存（OS）和无进展生存（PFS）曲线

4. 患者的OS：2008–2021年，应用BTKi、利妥昔单抗和蛋白酶体抑制剂为主新治疗方案的患者比例逐步提高，2008–2010年应用新治疗方案患者比例为50％，2011–2013年为42％，2014–2016年为70％，2017–2021年为93％。2015–2021年诊断WM 患者的OS较2008–2014年明显改善（中位OS时间：未达到对91个月，*P*＝0.048）（图2）。

**图2:**
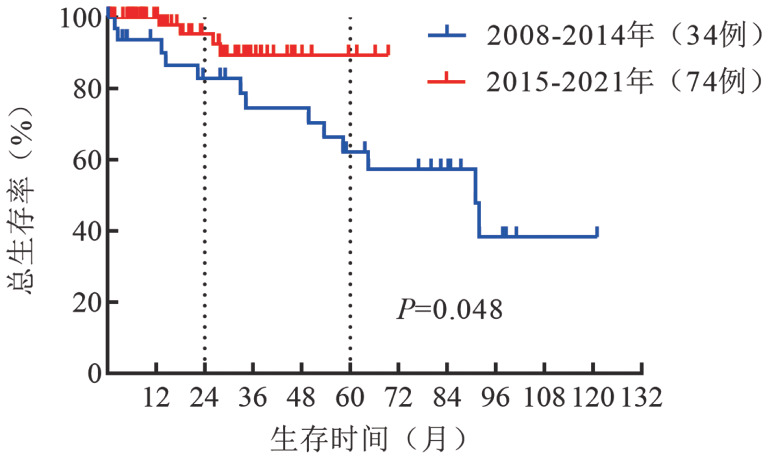
2008–2014年与2015–2021年诊断华氏巨球蛋白血症患者总生存曲线

5. 患者的PFS：2008–2021年，应用BTKi、利妥昔单抗和蛋白酶体抑制剂为主新治疗方案患者的2年PFS率为59％，5年PFS率为39％，而应用传统治疗方案患者2年PFS率为31％，5年PFS率为13％。新治疗方案与传统治疗方案相比，PFS明显改善（*P*＝0.040）（图3）。不同的新治疗方案中，以BTKi为主的靶向治疗2年PFS率为79％，以利妥昔单抗为主的免疫化疗2年PFS率为52％，其他治疗方案2年PFS率为44％，以BTKi为主的靶向治疗明显改善患者的PFS（*P*＝0.020）（图4）。

**图3:**
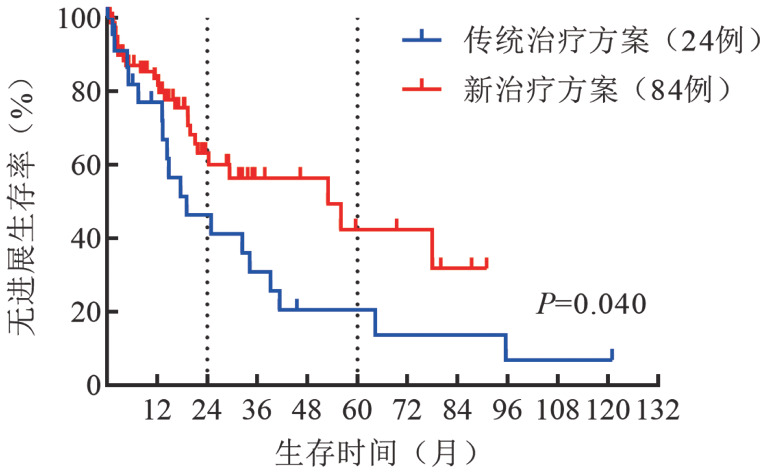
应用传统治疗方案与新治疗方案华氏巨球蛋白血症患者的无进展生存曲线

**图4:**
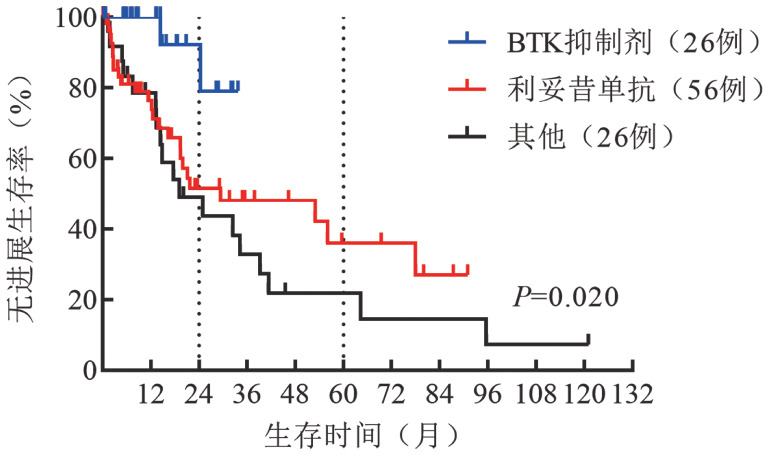
应用BTK抑制剂、利妥昔单抗和其他方案治疗的华氏巨球蛋白血症患者的无进展生存曲线

## 讨论

本中心在10余年WM诊断过程中，综合多种方法和多种类型标本，整体MYD88突变检测阳性率77％，其中1例患者为非MYD88^L265P^突变（MYD88^L273P^）。国外有研究采用AS-PCR检测CD19分选的骨髓，MYD88突变阳性率达93％～97％[7],[12]，而国内用类似方法检测MYD88阳性率为73％～94％[13]–[14]。本中心通过NGS方法检测MYD88阳性率88％，提示不同检测方法或标本可能影响MYD88检测阳性率。CXCR4有40余种突变类型，其中S338X突变常见，国外报道CXCR4检测阳性率为30％左右[15]，曹欣欣等[14]报道26％患者CXCR4突变，本中心32例NGS检测患者CXCR4突变阳性率仅16％，有待进一步扩大样本证实中国患者中CXCR4突变比例。Treon等[4]报道，在BTKi单药治疗中，MYD88^MUT^CXCR4^WT^患者预后佳，其次是MYD88^MUT^CXCR4^MUT^，MYD88^WT^CXCR4^WT^预后最差。CXCR4^WHIM^突变中，仅CXCR4无义突变与预后不良相关，而CXCR4移码突变与CXCR4^WT^患者的预后无明显差别[7]。鉴于MYD88和CXCR4突变在预后中的价值，如何提高WM初诊患者突变检测的阳性率和准确率值得进一步探究。

曹欣欣等[14]报道，中国MYD88^MUT^CXCR4^WT^患者中骨髓淋巴浆细胞比例7％，对应HGB为98 g/L，而MYD88^MUT^CXCR4^MUT^患者中骨髓淋巴浆细胞比例28％，对应HGB为75 g/L，提示骨髓淋巴浆细胞比例越高，贫血越严重。与Pivotal研究63例患者基线临床特征比较，本中心WM患者中位HGB为86 g/L，显著低于Pivotal研究（105 g/L），但骨髓中淋巴浆细胞比例为13％，明显低于Pivotal研究（60％），似乎与上述结论不符。然而，本中心患者骨髓淋巴浆细胞比例与曹欣欣等[14]报道的结果相符。此外，本中心患者淋巴结肿大比例较低、脾肿大比例较高，但与国内报道接近。两组患者年龄、性别、血清IgM水平、PLT、IPSS危险度分层的差异无统计学意义。

应用BTKi治疗WM推荐持续口服至不能耐受或疾病进展，Treon等[4]–[5]报道，BTKi能明显改善初治和经治WM患者的PFS，但长期口服BTKi患者的长期生存与真实世界数据的差异尚不清楚。Treon等[4]对2015年报道的63例口服BTKi单药经治WM患者随访了近5年，2021年报道了63例患者的5年PFS和5年OS[7]。我们以上述63例患者作为参照，与本中心108例患者的长期预后进行比较。结果表明，本中心患者的2年OS率（83％对96％）和5年OS率（67％对87％）均低于Pivotal研究患者。虽然此结果是与我中心历史数据比较，但值得注意的是，本中心108例患者中有26例（24％）首诊采用BTKi，有18例（17％）患者在应用其他方案病情进展后更换为BTKi。因此非BTKi和BTKi治疗患者实际长期OS的差距可能更大，提示BTKi单药可能给患者带来长期获益。

WM的治疗从以苯丁酸氮芥、CHOP、FC方案为主的传统治疗进展到以利妥昔单抗、蛋白酶体抑制剂、BTKi为主的新治疗[16]。值得欣喜的是，近10年来，本中心应用新治疗方案患者的比例逐步提高，从最初的50％提高到近年的93％，且2015–2021年WM患者的OS较既往明显改善，表明技术进步、新药研发和支持治疗改善为患者带来了福音。新旧治疗方案的比较也显示出新治疗方案明显改善PFS，但OS的改善还需更长的观察时间。WM发病率低，虽然是惰性淋巴瘤，但异质性高，根据2019年rIPSS诊断标准[17]，超高危患者5年OS率仅36％，10年OS率仅9％。2020年Treon等[12]推荐以MYD88突变和CXCR4突变的分子分型为基础选择合适的治疗方案。中国WM患者的最佳治疗方案及疗效还有赖于多中心临床研究和更多真实世界数据。
